# A comparative pharmaco-metabolomic study of glutaminase inhibitors in glioma stem-like cells confirms biological effectiveness but reveals differences in target-specificity

**DOI:** 10.1038/s41420-020-0258-3

**Published:** 2020-04-16

**Authors:** Katharina Koch, Rudolf Hartmann, Julia Tsiampali, Constanze Uhlmann, Ann-Christin Nickel, Xiaoling He, Marcel A. Kamp, Michael Sabel, Roger A. Barker, Hans-Jakob Steiger, Daniel Hänggi, Dieter Willbold, Jaroslaw Maciaczyk, Ulf D. Kahlert

**Affiliations:** 1 Neurosurgery Department, University Hospital Duesseldorf, 40225 Duesseldorf, Germany; 2 Institute of Complex Systems (ICS-6) Structural Biochemistry and JuStruct: Juelich Center for Structural Biology, Forschungszentrum Juelich, 52425 Juelich, Germany; 3 John van Geest Centre for Brain Repair and WT/MRC Cambridge Stem Cell Institute, Department of Clinical Neurosciences, University of Cambridge, CB2 0PY Cambridge, UK; 4 Institut für Physikalische Biologie, Heinrich Heine University Duesseldorf, 40225 Duesseldorf, Germany; 5 Neurosurgery Department, University Hospital Bonn, 53127 Bonn, Germany; 6 German Cancer Consortium (DKTK), Essen/Duesseldorf, Germany

**Keywords:** Cancer metabolism, Predictive markers, Translational research, CNS cancer, Cancer stem cells

## Abstract

Cancer cells upregulate anabolic processes to maintain high rates of cellular turnover. Limiting the supply of macromolecular precursors by targeting enzymes involved in biosynthesis is a promising strategy in cancer therapy. Several tumors excessively metabolize glutamine to generate precursors for nonessential amino acids, nucleotides, and lipids, in a process called glutaminolysis. Here we show that pharmacological inhibition of glutaminase (GLS) eradicates glioblastoma stem-like cells (GSCs), a small cell subpopulation in glioblastoma (GBM) responsible for therapy resistance and tumor recurrence. Treatment with small molecule inhibitors compound 968 and CB839 effectively diminished cell growth and in vitro clonogenicity of GSC neurosphere cultures. However, our pharmaco-metabolic studies revealed that only CB839 inhibited GLS enzymatic activity thereby limiting the influx of glutamine derivates into the TCA cycle. Nevertheless, the effects of both inhibitors were highly GLS specific, since treatment sensitivity markedly correlated with GLS protein expression. Strikingly, we found GLS overexpressed in in vitro GSC models as compared with neural stem cells (NSC). Moreover, our study demonstrates the usefulness of in vitro pharmaco-metabolomics to score target specificity of compounds thereby refining drug development and risk assessment.

## Introduction

Metabolic reprogramming of bioenergetic and biosynthetic processes is a key event in malignant transformation^1^. Tumor cells can maintain high proliferation rates by enhanced uptake and metabolism of glucose and glutamine (Gln) thereby fueling the two main anaplerotic pathways providing tricarboxylic acid (TCA) cycle intermediates to boost oxidative phosphorylation and biomass production^2^. Since glycolysis and glutaminolysis are dysregulated in neoplastic cells and tissues due to epigenetic and mutational changes^3–6^, they are compelling therapeutic targets in cancer therapy. However, the clinical application of glycolysis inhibitors remains challenging mainly due to adverse effects^7,8^. Alternative strategies of metabolic interference in cancer therapy have recently emerged including the restriction of glutaminolytic activity. Mitochondrial glutaminase (GLS) catalyzes the hydrolytic deamidation of Gln to glutamate (Glu) in the first step of glutaminolysis. Subsequently, Glu is either metabolized to the TCA cycle intermediate alpha-ketoglutarate (αKG), which is then used as a nitrogen-donor during the synthesis of several nonessential amino acids, or mediates redox homeostasis by increasing the production of the antioxidant glutathione (GSH). Since several oncogenes regulate GLS expression and many studies have shown that cancer cells are GLS-dependent^3,9,10^, GLS inhibitors (GLSi) (have been designed and evaluated in preclinical and clinical trials for brain tumors (trial ID: NCI-2018-00876)^11–13^. The specific and orally bioavailable small molecule inhibitor CB839 has been shown to effectively reduce viability, chemosensitivity, and induce apoptosis in several tumor entities including breast, ovarian, prostate, and lung cancer^14–17^. Furthermore, compound 968 (C968), another small molecule GLS inhibitor, has been reported to impair tumor cell clonogenicity, viability, and growth^18–23^. Glioblastoma (GBM), the most aggressive malignant brain tumor, expresses high levels of GLS however their dependency on Gln metabolism remains controversial^24^. Several studies report that GBMs depend on glutaminolysis to sustain cellular viability and TCA cycle anaplerosis^4,25,26^. However, others report that growth inhibition upon Gln withdrawal is cell line-specific and that resistant cell lines upregulate alternative metabolic routes to compensate for the lack of nutrient supply^27^. Here we analyze the effect of GLS inhibition on stem cell-enriched GBM in vitro models (GBM stem-like cells; GSCs), which have been suggested to be responsible for the emergence of therapy resistance and tumor relapse^28–31^. Accumulating evidences indicate that GSCs are characterized by distinct metabolic reprogramming and interfering in this may be a suitable strategy to combat those highly therapy resistance population of cells^32–34^. We assessed the therapeutic potential of two prominent GLSi on GSCs in vitro and validated GLS as a target with low adverse effects. Furthermore, the pharmaco-metabolic characterization of our in vitro models upon compound exposure highlights the potential of this technology for the risk assessment of drug candidates.

## Results

### GSC in vitro models recapitulate the glutaminase expression status of patient samples

**Fig. 1:**
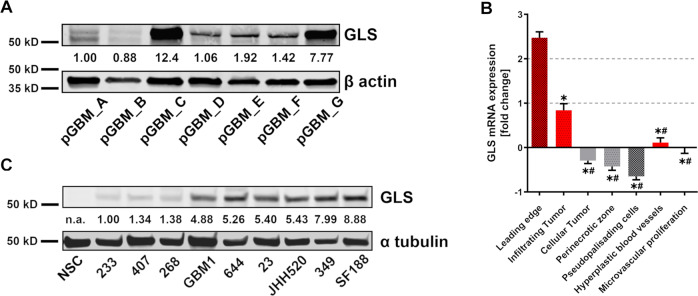
Primary GBM samples (in vivo) and stem-cell enriched GSC cultures (in vitro) show the same variation in GLS protein expression. **a** GLS protein expression was assessed in seven primary glioblastoma (pGBM) samples and compared with the loading control (β actin). **b** RNA sequencing data from the IVY glioblastoma project showing differential GLS expression in five tumor structures (Leading Edge, Infiltrating Tumor, Cellular Tumor, Microvascular Proliferation, and Pseudopalisading Cells around Necrosis), the hyperplastic blood vessels, and the microvascular proliferative region from 10 different tumors. *significantly decreased (*p* < 0.05) compared with Leading Edge. #significantly decreased (*p* < 0.05) compared with Infiltrating Tumor. Statistical significance was tested with a one-way ANOVA plus Bonferroni’s multiple comparisons test. The data are represented as mean ± SD (*n* = 19–122, see “Methods” section for details). **c** GLS protein expression in NSCs and nine GSC cultures was assessed with immunoblotting and compared with the loading control (α tubulin). GLS glutaminase, n.a. not available, NSCs neural stem cells, pGBM primary glioblastoma.

In order to assess the relevance of GLS in human GBM tumors, tumor bulk samples of seven primary GBMs were homogenized and subjected to immunoblotting for GLS protein. GLS was clearly expressed in all GBMs although expression varied markedly between tumors (Fig. 1a). That suggests, that the susceptibility of a patients GBM to GLS inhibition varies as well. Publicly available mRNA expression data showed that GLS is markedly overexpressed in the leading edge of the tumor and infiltrating tumor tissue (Fig. 1b) and it is thought that these tumor compartments promote GSC enrichment through a mechanism called epithelial–mesenchymal (EMT)-like transition^35^. Concisely, GLS has been reported to regulate the expression of EMT markers in colorectal cancer^36,37^. To assess GLS expression in stem cell-enriched GBM cultures, we performed immunoblotting for GLS protein in nine GSC cultures and compared the expression level to non-tumorigenic neural stem cells (NSCs) (Fig. 1c). Strikingly, we observed lowest GLS expression in NSCs and identified significant variations between the nine GSC cultures. Hence, we decided to study GLS suppression in six different GSC neurosphere cultures, three GLS-low (233, 407, 268) and three GLS-high (GBM1, JHH520, SF188) expressing ones. Importantly, our selected in vitro GSC models represent the physiological variation of GLS expression observed in tumor tissue therefore showing pathophysiological relevance (Fig. 1a). Verified by pixel densitometry analysis, the variation in GLS protein observed in the different primary tumors is similar to the variation in GLS expression observed between the different GSC in vitro models. For both protein analyses (Fig. 1a, c), the same experiment conditions were used and equal amounts of total protein were loaded.

### Susceptibility to pharamcological glutaminase inhibition correlates with elevated GLS expression

**Fig. 2:**
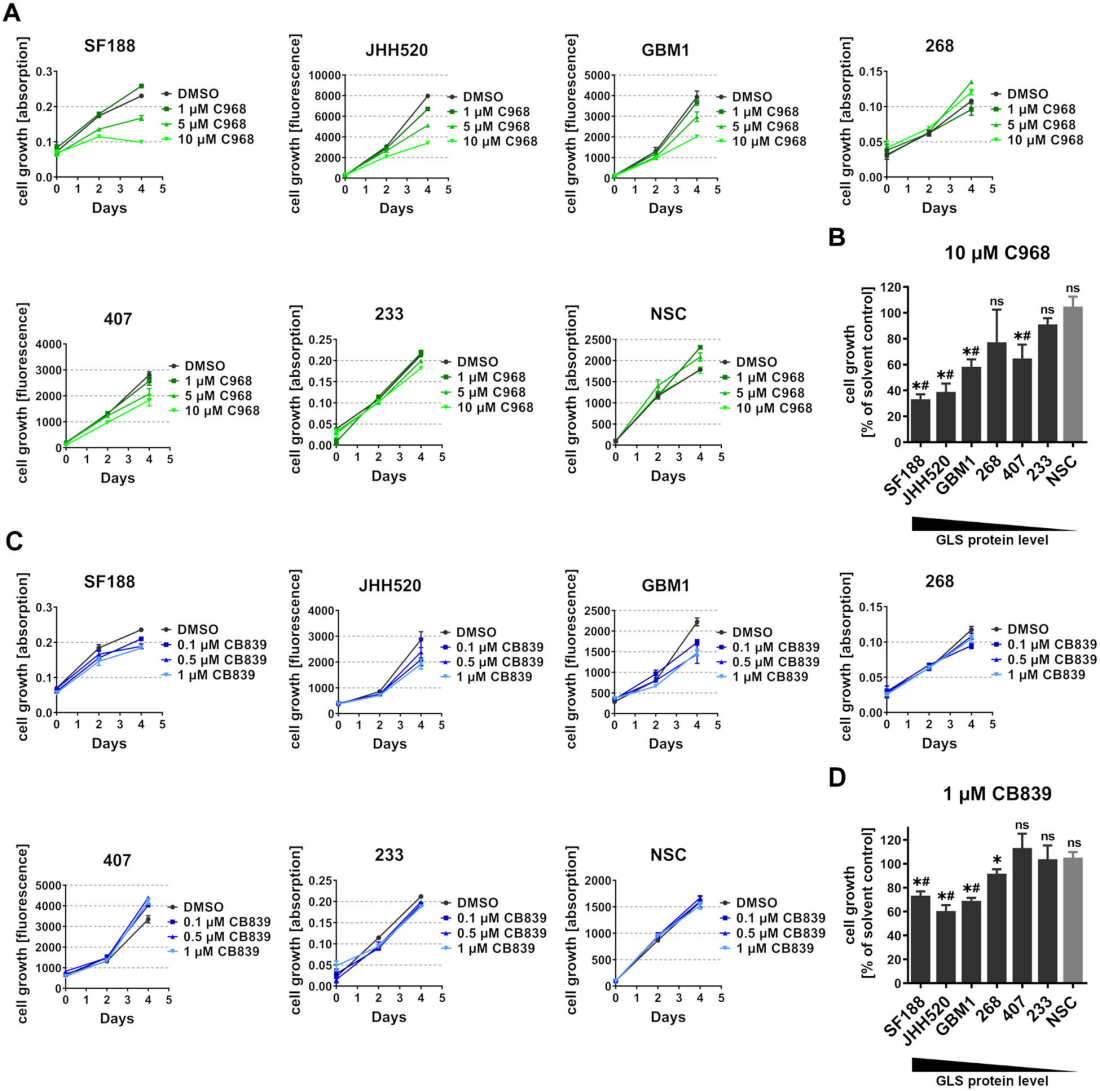
Sensitivity to GLS inhibition correlates with high GLS protein expression. Both GLS-high GSCs (SF188, JHH520, GBM1), GLS-low GSCs (233, 407, 268), and NSCs were exposed to increasing concentrations of GLS inhibitors C968 (**a**) and CB839 (**c**). The growth of GSCs and NSCs upon 10 µM C968 (**b**) and 1 µM CB839 (**d**) treatment for 48 h was compared with the growth under the respective solvent controls (DMSO). The growth under solvent control was set to 100%. *, significant compared with the solvent control. ns, not significant compared with the solvent control. #, significant compared with the inhibitor-effect in NSCs. Cell lines were sorted from highest to lowest GLS protein expression level (*n* = 3–4, specified in methods). Statistical significance was calculated with unpaired *t*-tests. All data are depicted as mean ± SD (*p < 0.05). d days, DMSO dimethylsulfoxid, n.s. not significant, NSC neural stem cells.

To assess the effect of GLS inhibition on tumorigenic GSCs and estimate possible side effects on non-tumorigenic stem cells, we treated highly clonogenic neurosphere cultures derived from human fetal cortical tissue (neural SC; NSC) with the small molecule GLSi C968 and CB839 and compared them to GLS-high (SF188, JHH520 and GBM1) and GLS-low (233, 407, and 268) expressing GSCs. Fetal NSCs are a commonly used surrogate for non-tumorigenic stem cells in brain tumor research^38–41^. Cell growth of GSC neurospheres and NSCs treated with increasing concentrations of C968 (Fig. 2a, b), CB839 (Fig. 2c, d), or vehicle (DMSO) was assessed after two and four days of incubation. In SF188, JHH520 and GBM1 cells, treatment with both C968 and CB839 induced a dose-dependent suppression of cell growth. On the contrary, 233, 407, and 268 showed no effects to GLS inhibition except for a moderate response in 407 to C968 and 268 to CB839 exposure. Non-tumorigenic NSCs were resistant to both C968 and CB839 treatment.

### 2.3. Glutaminase inhibition selectively impairs the stemness phenotype of GLS-high expressing cultures

**Fig. 3:**
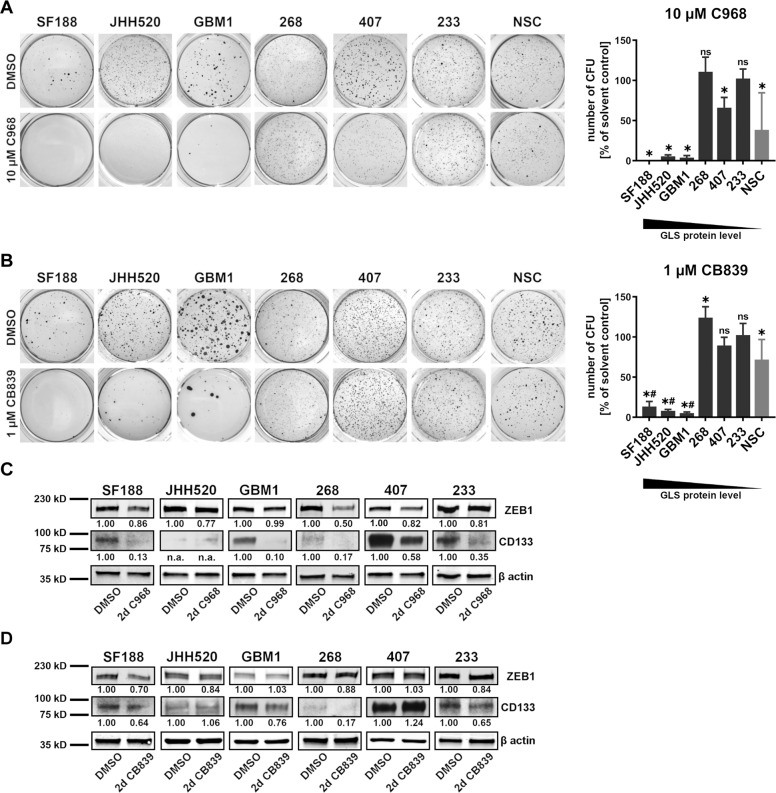
Treatment with C968 and CB839 eradicates tumor initiating cells in GLS-high expressing cultures. GSC cultures and NSCs were treated with 10 µM C968 (**a**), 1 µM CB839 (**b**), or solvent control (DMSO). The number of colonies counted in cells treated with the solvent control (DMSO) was set to 100%. Displayed are the number of colonies (CFU) in the inhibitor-treated conditions compared with the colonies in the respective solvent controls (% of solvent control). *, significant compared with the solvent control. ns, not significant compared with the solvent control. #, significant compared with the inhibitor-effect in NSCs. Representative pictures of NBT stained colonies and quantifications of three soft agar colony formation assays are shown (*n* = 3–6, specified in methods). Statistical significance was calculated with unpaired *t*-tests. Immunoblotting of stemness markers ZEB1 and CD133 in GSC cultures treated for 48 h with 10 µM C968 (**c**), 1 µM CB839 (**d**) or vehicle (DMSO). GSC cultures were sorted according to their GLS expression level from highest (SF188) to lowest (233) expression (loading control = β actin). All data are depicted as mean ± SD (*p < 0.05). d days, DMSO dimethylsulfoxid, n.a. not available, NBT nitro blue tetrazolium chloride, n.s. not significant, NSC neural stem cells.

Since GSCs maintain their self-renewal ability they are capable of growing colonies out of single cells. Therefore, we tested the in vitro clonogenicity of GSCs upon treatment with 10 µM C968 (Fig. 3a), 1 µM CB839 (Fig. 3b), or vehicle and compared them to non-tumorigenic NSCs. Consistent with the results from the growth assays, SF188, JHH520, and GBM1 cells showed markedly diminished clonogenic capacity, while the in vitro clonogenicity of GLS-low expressing cells (233, 407, and 268) was not affected except for a moderate reduction in 407 cells treated with C968. We can therefore directly correlate resistance to GLS inhibition with low protein expression levels (Fig. 1c). Consistent with our results, both pharmacological and genetic suppression of GLS protein caused impaired clonogenic capacity in different tumor entities^17,23,42,43^. Although reaching statistical significance, the in vitro clonogenicity was barely reduced in NSCs treated with 1 µM CB839 and moderately reduced by 10 µM C968 treatment (Fig. 3a, b). The observed effects were much less severe than observations in previous studies testing Gln analogs or genetic GLS suppression in non-tumorigenic NSCs^44,45^. To our knowledge, our study is the first risk assessment of targeted pharmacological GLS inhibition in NSCs. Again, the superiority of CB839 as a GLSi becomes obvious, showing less side effects in NSCs. Previous research directly correlated the clonogenic capacity of GSCs with the expression of GSC stemness marker prominin (CD133) and EMT activator ZEB1^31,35,46–48^. Immunoblotting analyses should show whether the impaired clonogenicity we observed in treated GLS-high expressing GSCs (SF188, JHH520, GBM1) coincides with reduced CD133 or ZEB1 expression. Both C968 (Fig. 3c) and CB839 (Fig. 3d) treatment mildly reduced ZEB1 expression both in sensitive (GLS-high) and insensitive (GLS-low) GSCs. Furthermore, CD133 expression was dramatically reduced by C968 and moderately reduced by CB839 treatment independent of GLSi sensitivity. Since we observed similar effects in both sensitive and insensitive GSCs, our results clearly show that the degree of stemness in GBMs cannot be exclusively estimated by the expression levels of single markers like CD133 or ZEB1, but should rather be defined by phenotypical readouts like the in vitro clonogenicity.

### Glutaminase inhibition causes cell cycle arrest in sensitive GLS-high expressing GSCs

**Fig. 4:**
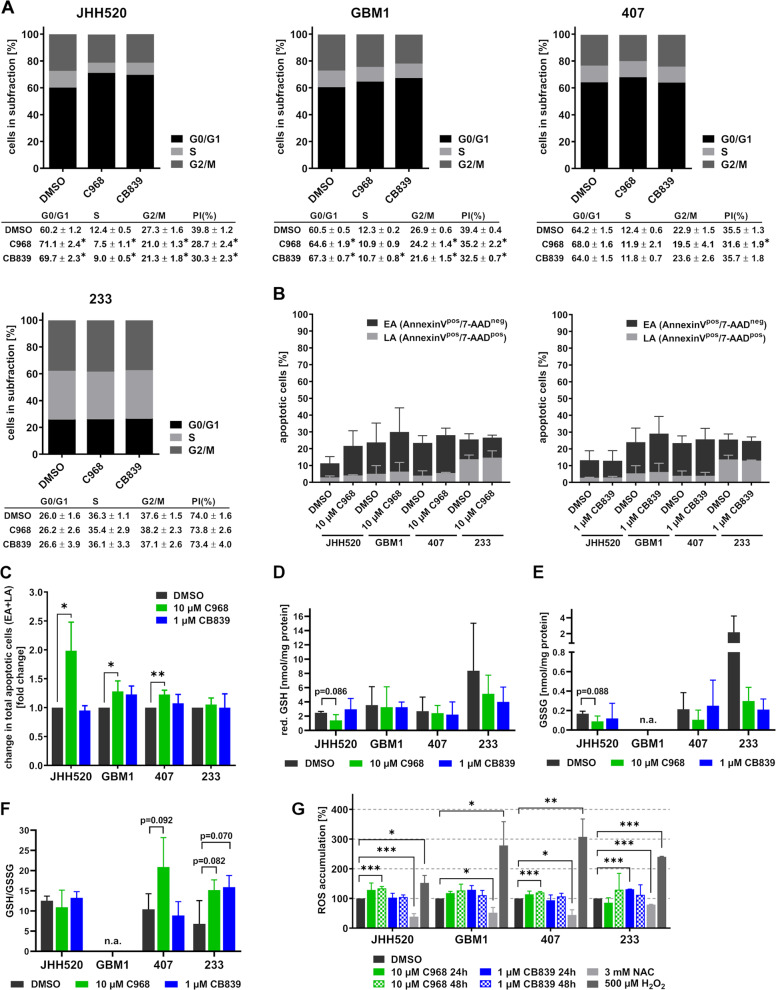
CB839 selectively causes cell cycle arrest in GLS-high expressing cultures without inducing apoptosis or oxidative stress. **a** Percentage of cells in quiescent (G0/G1), synthesis (S), or mitosis (G2/M) phase after treatment with 10 µM C968, 1 µM CB830, or vehicle (DMSO) for 48 h. The proliferation index (PI = (S + G2/M)/(G0/G1 + S + G2/M) x 100%) was calculated to visualize the percentage of proliferative cells. Statistical significance was tested with two-way ANOVA (*n* = 3). **b** The percentages of early apoptotic (EA, AnnexinV^pos^, 7-AAD^neg^) and late apoptotic (LA, AnnexinV^pos^, 7-AAD^pos^) cells in GSC cultures treated for 48 h with 10 µM C968 and 1 µM CB839 were assessed with flow cytometry. **c** Fold changes in total apoptotic cells (EA + LA) of C968- and CB839-treated GSCs compared with cultures treated with DMSO (*n* = 3 for C968, *n* = 4 for CB839). Reduced (GSH) [nmol/mg protein] (**d**) and oxidized (GSSG) [nmol/mg protein] (**e**) glutathione was measured in GSCs treated with 10 µM C968, 1 µM CB830, or vehicle (DMSO) for 48 h. **f** The ratio of reduced (GSH) and oxidized (GSSG) glutathione in GSCs treated with 10 µM C968, 1 µM CB830, or vehicle (DMSO) for 48 h (*n* = 3). **g** ROS accumulation was measured via DCFDA oxidation over 24 h and 48 h in cells treated with either 10 µM C968, 1 µM CB830, or DMSO. Exposure to 1 mM NAC was used as the negative and exposure to 500 µM H_2_O_2_ as the positive control (*n* = 3). If not specified, statistical significance was calculated with unpaired *t*-tests. All data are depicted as mean ± SD (*p < 0.05, **p < 0.01, ***p < 0.001). DMSO dimethylsulfoxid, GSH glutathione, n.a. not available, NAC N-acetylcysteine, n.s. not significant, ROS reactive oxygen species.

Since functional glutaminolysis is a prerequisite for a variety of cellular processes, we assessed apoptosis induction, reactive oxygen species (ROS) accumulation, GSH concentrations and GSH/GSSG ratios, and the cell cycle state in GSCs treated with 10 µM C968 or 1 µM CB839. For those mechanistic studies we chose two GLS-high (JHH520 and GBM1) and two GLS-low (407 and 233) expressing GSC cultures. In GLS-high expressing cells, treatment with both inhibitors significantly increased the percentage of cells in quiescent G0/G1 phase and reduced the percentage of cells in the proliferative S and M/G2 phases (Fig. 4a). However, treatment with C938 and CB839 did not significantly alter the cell cycle of GLS-low expressing 233 or 407 cells. Furthermore, we calculated the proliferation index as a measure of cells in proliferative cell cycle phases (PI = (S + G2/M) / (G0/G1 + S + G2/M) x 100%). We observed a significant decrease of the PI in JHH520 (−28%), GBM1 (−11%), and 407 (−11%) upon C968 treatment and in JHH520 (−24%) and GBM1 (−18%) cells upon CB839 treatment. In lowest GLS expressing 233 cells neither C968 nor CB839 affected the PI. Furthermore, we co-stained GSCs treated with GLSi for apoptotic cells with AnnexinV and 7-AAD. FACS measurements revealed no significant increase in the percentages of early apoptotic (EA, AnnexinV^pos^, 7-AAD^neg^) or late apoptotic (LA, AnnexinV^pos^, 7-AAD^pos^) cells upon C968 or CB839 treatment (Fig. 4b). However, when we normalized the percentages of total apoptotic cells (EA + LA) in the treatment conditions to the respective solvent controls we observed a significant increase in total apoptotic cells by 1.99-fold in JHH520, 1.28-fold in GBM1, and 1.23-fold in 407 cells treated with C968, but saw no effect in 233 cells (Fig. 4c). CB839 treatment did not significantly induce apoptosis in any of the cell lines. Since the GLS product Glu is crucial for the synthesis of the antioxidant GSH, we assessed whether GLS inhibition alters the concentrations of reduced (GSH) or oxidized (GSSG) glutathione or affects the GSH/GSSG ratio. Decreased GSH/GSSG ratios are a measure of oxidative stress in vitro and in vivo^49^. Except for a nearly significant reduction of GSH (*p* = 0.086) and GSSG (*p* = 0.088) concentrations in C968-treated JHH520 cells, we observed no significant effects on the GSH or GSSG concentrations (Fig. 4d, e). Interestingly, the GSH/GSSG ratio was even increased in insensitive GLS-low 407 (*p* = 0.092 for C968) and 233 (*p* = 0.082 for C968 and *p* = 0.070 for CB839) cells treated with GLSi (Fig. 4f). In GBM1 cells, we could not detect GSSG and thus could not calculate the GSH/GSSG ratio. This is not uncommon, since reduced GSH makes up on average 98% of the total GSH content^49^. Since alterations in antioxidant levels affect the intracellular clearance of ROS, we assessed ROS accumulation after treatment with 10 µM C968 or 1 µM CB839 for 24 and 48 h. Except for a 30% increase in ROS accumulation in 233 cells treated with CB839 for 24 h, CB839 treatment did not induce a significant increase in intracellular ROS (Fig. 4g). Furthermore, 48 h C968 exposure increased ROS levels in GLS-high JHH520 (34%) and GLS-low 407 (20%) cells. The ROS inhibitor N-Acetyl-L-cysteine (NAC) significantly reduced and H_2_O_2_ significantly increased ROS accumulation. Consistent with our previous findings, these results further emphasize the selective mode of action of GLSi on cells with abundant GLS protein expression, since especially the observed effects on viability, clonogenicity, cell cycle, and apoptosis were most pronounced in GLS-high expressing cells.

### Glutaminase inhibition with CB839 targets the anabolism of GSCs by diminishing the influx into the TCA cycle

**Fig. 5:**
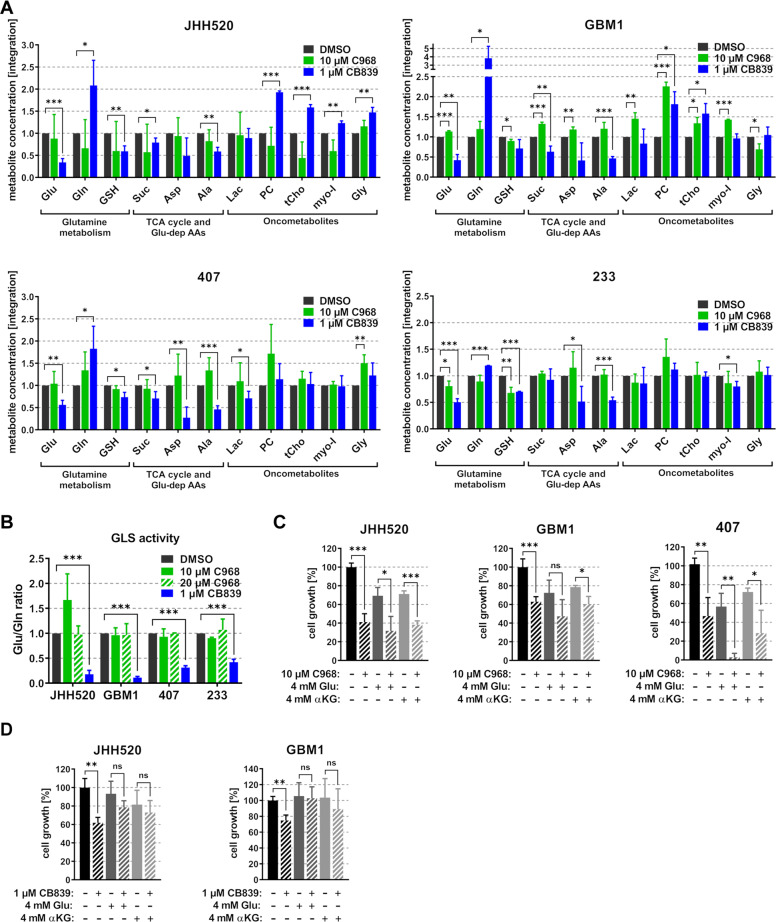
CB839 effectively blocks glutaminolysis and reduces the influx into the TCA cycle. **a** Relative metabolite concentrations of GSC cultures treated for 48 h with 10 µM C968, 1 µM CB839, or DMSO were assessed with HR ^1^H NMR spectroscopy of the water-soluble metabolome (*n* = 3). **b** The GLS activity after 48-h treatment with 10 µM C968, 20 µM C968, 1 µM CB839, or DMSO is depicted by the product (Glu) to educt (Gln) ratio of GLS (*n* = 3). Cell growth of GSCs treated with 10 µM C968 (**c**), 1 µM CB839 (**d**), or DMSO for 48 h either in the presence of medium only, 4 mM Glu, or 4 mM αKG (*n* = 3). For all assays, statistical significance was calculated with unpaired *t*-tests. All data are depicted as mean ± SD (*p < 0.05, **p < 0.01, ***p < 0.001). AA amino acids, αKG alpha ketoglutarate, Ala alanine, Asp aspartate, CFU colony forming units, DMSO dimethylsulfoxid, Gln glutamine, Glu glutamate, Gly glycine, GSH glutathione, Lac lactate, myo-I myo-inositol, n.s. not significant, PC phosphocholine, ppm parts per million, Suc succinate, TCA tricarboxylic acid, tCho total choline, tCre total creatine.

To see whether metabolic changes cause the phenotypic effects of GLS inhibition, we used high resolution proton magnetic resonance spectroscopy (HR ^1^H NMR) to analyze intracellular metabolites of JHH520, GBM1, 407, and 233 cells treated with 10 µM C968 or 1 µM CB839. Surprisingly, only CB839 treatment induced the anticipated changes in GSC metabolites. Blockade of GLS activity by CB839 led to a significant reduction of the enzyme product (Glu), accumulation of the educt (Gln), and decreased the Glu/Gln ratio in all four cell lines (Fig. 5a, b). This metabolic phenotype has been validated in several in vitro and in vivo studies as a reproducible measure of GLS inhibition^14,50,51^. Strikingly, upon treatment with C968 we neither observed reduced product (Glu) concentrations nor increased educt (Gln) concentrations, except for a moderate Glu reduction in 233 cells. Even in most sensitive JHH520 cells, C968 treatment did not decrease the Glu/Gln ratio. Since Wang et al. showed efficient GLS inhibition with 20 µM C968 in hepatocellular carcinoma cells which do not respond to 10 µM C968^20^, we additionally measured the GLS activity in GSC cultures treated with 20 µM C968. However, treatment of our GSC cultures with 20 µM C968 did not alter the Glu/Gln ratio and therefore did not inhibit the GLS activity. Furthermore, CB839, but not C968, reduced intracellular concentrations of the TCA cycle intermediate succinate (Suc) and Glu-dependent amino acids aspartate (Asp) and alanine (Ala) in all four cell lines. We further analyzed levels of known oncometabolites upon treatment with C968 and CB839 to see whether GSCs regulate other metabolic pathways that may compensate for glutaminolysis inhibition. In sensitive (JHH520, GBM1) but not insensitive (407, 233) cells CB839 treatment increased choline metabolism leading to elevated levels of phosphocholine and total choline. In contrast, C968 treatment had no consistent effect on GSC oncometabolites. Since we observed reduced Suc, Asp, and Ala levels upon CB839 treatment, we hypothesized that insufficient supply of TCA cycle intermediates after GLS inhibition disturbs the anabolism of GSCs causing a decline in the GSC pool. To test this hypothesis, we tried to rescue the anti-growth effect of C968 and CB839 by addition of either 4 mM Glu or 4 mM αKG. The C968 treatment could neither be rescued by Glu nor αKG (Fig. 5c). In contrast, the anti-growth effect of CB839 in sensitive JHH520 and GBM1 cells could be efficiently rescued by both addition of Glu and αKG (Fig. 5d), indicating that CB839 eradicates GSCs by diminishing TCA-cycle dependent processes necessary for GSC maintenance and cell cycle progression. Consistent with our results, previous studies reported that the maintenance of cancer stem-like cells depends on a high flux rate through the TCA cycle^52,53^ and that cell cycle progression in cells is limited by the availability of biosynthetic precursors from the TCA cycle^54,55^. Our ^1^H NMR data, clearly show that C968 fails to suppress glutaminolysis, since we neither observed a reduction in the GLS product Glu, nor any downstream products of Glu such as Suc, Asp, or Ala upon C968 treatment (Fig. 5a, b). Interestingly, CB839 also induced metabolic changes in insensitive 407 and 233 cells. However, the effects were less marked than those observed in GLS-high expressing JHH520 and GBM1 cells. We hypothesize that due to the low GLS baseline expression, 407 and 233 cells are less dependent on functional GLS.

## Discussion

Functional glutaminolysis is crucial for the bioenergetic and biosynthetic homeostasis especially in proliferative and invasive cancer cells. Here we show that maintenance of highly aggressive GSCs markedly depends on intracellular Glu synthesis by GLS and that this dependency strongly correlates with GLS protein expression. We show that GLS is expressed in GBM tumors and its level of expression greatly varies among patients (Fig. 1a). In a previous study on matching control brain and tumor samples from GBM patients, it was further shown that tumors express elevated GLS protein levels^26^. Moreover, increased GLS expression has been correlated with higher grade brain cancers, shortened patient survival, and temozolomide (TMZ) resistance^24^. In this manuscript we show elevated GLS transcription in the invasive front of GBMs, known to contain highly invasive GSCs^35^. Previous publications reported that genetic and pharmacological inhibition of GLS attenuates stemness properties in hepatocellular, colorectal, and prostate cancer^21,23,43^. Furthermore, our group showed that the prominent route of anti-GSC therapy using γ-secretase inhibitor MRK003 targets GSC growth, in part by reducing intracellular Glu as a consequence of GLS inhibition^56^.

Here we correlate the resistance to GLS inhibition with reduced protein levels of GLS showing strong phenotypical consequences in high expressing GSC cultures (SF188, JHH520, GBM1) but only mild in low expressing GSCs (233, 407, 268) or non-neoplastic NSCs (Figs. 2, 3). This is in line with similar observations in triple-negative breast cancer (TNBC)^57^. Furthermore, the observed anti-stemness effect of GLS inhibition has been reported by several studies highlighting impaired clonogenicity as a major phenotype of pharmacological and genetic GLS suppression^23,43,58^.

Recent studies reported that targeted GLS inhibition further causes cell cycle arrest in prostate and non-small cell lung cancer^19,43^. This is consistent with our results showing cell cycle arrest in G0/G1 and a reduced fraction of cells in S- and M-phase after treatment with C968 (JHH520, GBM1, 407) and CB839 (JHH520 and GBM1) (Fig. 4). Again, especially the effectiveness of CB839 treatment correlated with the GLS expression level. Clonogenic GSCs make up just around 10% of our cultures (max. 10% of plated cells form colonies, Fig. 3), therefore the observed effects on cell cycle could explain the profound effects on clonogenicity if we assume that GSCs are especially affected by GLS inhibition.

Off-target effects are a common problem of many prominent cancer drug candidates in clinical trials^59^. Given the increasing attention of GLSi in various cancer trials, we sought to probe the target specificity of two leading GLSi compounds with the focus on their functional effects on malignant and nonmalignant stem cells. We chose C968 given its reproducibly reported therapeutic potential in many cancer studies including our own^19,21,22^, and CB839, as one of the leading clinical GLSi compounds in oncology (trial IDs: NCI-2018-00876, NCI-2019-01365, NCI-2019-00572). To our knowledge, no study has explicitly addressed the metabolic consequences of these compounds in functional assays which score their therapeutic effects preferentially as a consequence of effective target suppression. Surprisingly, our pharmaco-metabolic studies revealed that C968 treatment neither increased intracellular Gln, nor did it reduce the concentrations of Glu, Glu-dependent amino acids (Ala, Asp) or the TCA cycle intermediate Suc (Fig. 5), all validated metabolic indicators for successful GLS suppression^51,57,60^. This suggests that the phenotypes observed upon C968 treatment (Figs. 2, 3) cannot be explained by glutaminolysis suppression. This is supported by our observations that the effect of C968 treatment could neither be rescued by addition of Glu nor by replenishing the TCA cycle with αKG. This strongly suggests that C968 does not affect the enzymatic activity of GLS in GSCs. On the other hand, since sensitivity towards C968 treatment positively correlates with elevated GLS protein expression levels (Figs. 2, 3), the effects cannot solely be explained by non-specific cytotoxicity and application of metabolic flux analyses with isotope-labeled Gln are needed to pin-point the mode of action of this drug candidate.

In contrast, CB839 effectively reduced intracellular Glu concentration while causing accumulation of Gln (Fig. 5). Furthermore, CB839 diminished the influx into the TCA cycle and interfered with GSC anabolism by markedly reducing the levels of Suc, Ala, and Asp. These observations are in line with several pharmaco-metabolic studies showing Gln accumulation and decreased Glu, GSH, and Asp concentrations upon CB839 treatment^14,61^. Previous studies have reported that cancer stem-like cells depend on a high flux rate through the TCA cycle^52,53^, suggesting that CB839 treatment diminishes the GSC population by reducing the influx of Glu into the TCA cycle. Indeed, both addition of Glu and αKG effectively rescued the phenotype caused by CB839 treatment (Fig. 5). This is in line with previous research showing that upregulation of αKG-dependent Gln metabolism and increased GLS expression promotes the maintenance of cancer stem cells through various mechanisms^26,62–65^. Furthermore, Gln metabolism promotes the maintenance of stemness through elevating the synthesis of GSH and maintenance of a balanced redox homeostasis^23,66,67^. However, we could neither detect significant accumulation of ROS nor decreases in the GSH/GSSG ratios in GSCs treated with CB839 (Fig. 4). Therefore, we conclude that CB839 predominantly diminishes the GSC pool by disrupting the influx of Glu into the TCA cycle, thereby limiting the bioenergetic and biosynthetic supply. Several studies describe a checkpoint in the late G1 phase of the cell cycle where the progress into S phase depends on the availability of precursors for nucleotide biosynthesis^54,55^. Therefore, reduced influx into the TCA cycle could explain the observed cell cycle arrest in G0/G1 phase upon treatment with CB839.

Taken together, our study clearly shows the potential of in vitro pharmaco-metabolomics for therapy efficacy scoring and risk assessment of compounds. Focusing on GBM and their therapy-resistant stem cell subpopulation, we further highlight the relevance of GLS as a druggable and promising therapeutic target in our need to improve the management of GBM therapy resistance and tumor relapse. Although new platforms for computational drug target discovery using molecular and cellular data of tumor material enable high throughput drug design and therapy resistance prediction^68^, functional assays to biologically confirm computational biology findings are fundamental for the translational value of drug development and toxicology risk assessment. We found that CB839 significantly outperforms C968 in terms of enzymatic inhibitory potential and would be the preferred pharmacologic intervention when aiming at targeting glutaminolysis. CB839 shows effective GLS inhibition at low µM concentrations (1 µM) whereas even high concentrations of C968 (20 µM) do not affect GLS enzymatic activity. Our in vitro studies with non-transformed cells highlight the potential of CB839 as a cancer-specific precision treatment. Furthermore, our lab is aiming to improve the cancer cell specificity of CB839 and further reduce off-target effects by preferentially directing the delivery of the substance to cancer cells using nanotechnology engineering in a similar approach as recently described^69^.

## Materials and methods

### Cell cultures and primary tissue specimen

Glioma cell line JHH520 was generously provided by G. Riggins (Baltimore, USA), GBM1 by A. Vescovi (Milan, Italy), cell lines 23, 233, 268, 349, and 407 by M.S. Carro (Freiburg, Germany), cell line SF188 by E. Raabe (Baltimore, USA), and cell line NCH644 (644) by C. Herold-Mende (Heidelberg, Germany). GSC neurospheres were cultured in DMEM w/o pyruvate (Gibco, #11965092, Thermo Fisher Scientific, USA), 30% Ham’s F12 Nutrient Mix (Gibco, #11765047), 2% B27 supplement (Gibco, #17504044), 20 ng/ml human bFGF (Peprotech, #AF-100-18B, USA), 20 ng/ml human EGF (Peprotech, #AF-100-15), 5 µg/ml Heparin (Sigma, #H0878, Merck KGaA, Germany), and 1× Anti-Anti (Gibco, #15240096). All cells were cultured at 37 °C and 5% CO_2._ Cell lines were regularity tested for mycoplasma contamination and STR analyses were performed to guarantee authenticity and purity. Human fetal cortical tissue was collected in Cambridge UK under full ethical approval and sent to us where it was then transferred into cell culture after mechanic dissociation of cells. The cultures were enriched for NSCs by propagation in the above described neurosphere medium. Neurospheres were passaged by mechanical chopping with a McIlwan Tissue Chopper (Campden Instruments, UK) every week. The study was conducted in accordance with the Declaration of Helsinki, and the protocol was approved by the Ethics Committee of the Medical Faculty of the Heinrich-Heine University (Study ID #5206). Primary GBM tumor samples were derived from the operating theater at the department of neurosurgery (Duesseldorf, Germany) and were snap frozen in liquid nitrogen until the preparation of lysates was undertaken. All subjects gave their informed consent for inclusion before they participated in the study. The study was conducted in accordance with the Declaration of Helsinki, and the protocol was approved by the Ethics Committee of the Medical Faculty of the Heinrich-Heine University (#2019-484-FmB).

### GLS inhibitors

For GLS inhibition we used the small molecule inhibitors C968 (5-[3-bromo-4-(dimethylamino)phenyl]-2,3,5,6-tetrahydro-2,2-dimethyl-benzo[a]phenanthridin-4(1H)-one, Merck, Germany, #352010) and CB839 (N-[5-[4-[6-[[2-[3-(trifluoromethoxy)phenyl]acetyl]amino]-3-pyridazinyl]butyl]-1,3,4-thiadiazol-2-yl]-2-pyridineacetamide, Cayman Chemicals, USA, #22038). Stock solutions were prepared in dimethylsulfoxid (DMSO) and stored at −20 °C.

### Western blotting

Cell lysates were electrophoretically separated by SDS PAGE and transferred onto nitrocellulose membranes as described previously^70^. Primary antibodies against CD133 (1:100, Miltenyi, Germany, #W6B3C1), SOX2 (1:1000, Cell Signaling Technology, UK, #L1D6A2), GLS (1:1000, Abcam, UK, #ab93434), ZEB1 (1:2000, Sigma, #HPA027524), β actin (1:5000, Cell Signaling Technology, #4970) and α-tubulin (1:10,000, Sigma, #T9026) were incubated overnight at 4 °C in 5% milk powder in Tris-buffered saline with 0.1% Tween-20 (TBST). The secondary antibodies goat-anti-rabbit IRDye800CW (1:10,000, LI-COR, USA, #926-32211), goat-anti-mouse IRDye680RD (1:10000, LI-COR #926-68070), and goat anti-mouse-HRP (1:10,000, Jackson ImmunoResearch, UK, #111–035–003) were diluted in 5% milk powder in TBST and incubated for 1 h at room temperature. Chemiluminescent signals were detected on a film-based system using chemiluminescent substrates (Thermo Scientific, #34096). Fluorescence-labeled antibodies were detected with a LI-COR Odyssey CLx Imager (LI-COR). Densitometry was performed with supplied software from LI-COR or ImageJ software^71^. For protein analysis of primary tumor samples, tissues were homogenized in RIPA lysis buffer using a 1 ml Dounce Homogenizer. Lysates were then incubated for 45 min on ice and centrifuged at 13,000 rpm to yield the cleared lysate. For the immunoblot in Fig. 1c, we rearranged the individual lanes from lowest to highest GLS expression to improve visualization of the different expression patterns. All unprocessed pictures can be found in supplementary fig. 1. The remaining blots (Figs. 1, 3c, d) were not cut vertically. For all western blots, individual genes were tested on the same samples on the same membrane.

### Dual-phase metabolite extraction

Water soluble metabolites were extracted as previously described^70,72,73^. In brief, a minimum of 5 × 10^6^ cells per sample were harvested, washed with PBS, and extracted with the dual-phase methanol/chloroform/water (1:1:1, v/v/v) method. The cells were washed twice with 5 ml ice-cold 0.9 mM NaCl, resuspended in 850 µl ice-cold ddH_2_O and transferred into pre-chilled glass tubes. After addition of 4 ml of ice-cold methanol the tubes were vortexed vigorously and incubated on ice for 15 min. Then 4 ml of ice-cold chloroform was added, vortexed, and incubated for 10 min on ice. Finally, 3.15 ml of ice-cold ddH_2_O was added, vortexed, and incubated overnight at 4 °C. The samples were centrifuged for 30 min at 4 °C and 4500 rpm. The upper water-methanol phase was separated and incubated for 10 min with 10 mg Chelex® 100 resin (Sigma, #C7901) on ice. The samples were filtered through a 70 µm mesh and the methanol was evaporated for 1 h at 30 °C in a vacuum concentrator. Finally, the samples were frozen at −80 °C, lyophilized and stored at −20 °C until spectroscopy measurement.

### NMR data acquisition and processing

Prior to ^1^H NMR analysis, the lyophilisates were resuspended in 20 mM phosphate buffer (pH 7.0) containing 10% D_2_O and 3-(Trimethylsilyl) propionic acid (TSP; Lancaster Synthesis, USA) as an internal standard as described previously^70^.

One-dimensional ^1^H NMR spectra were acquired with a Bruker AVANCE III HD 700 spectrometer (Bruker, USA) equipped with a 5 mm HCN TCI cryo-probe operating at 700 MHz (16.4 Tesla). The ^1^H NMR data were obtained using excitation sculpting for water suppressing and the following acquisition parameters: 25 °C sample temperature, 9800 Hz sweep width, 256 transients with 32K time-domain data points were accumulated with a repetition time of 3.2 s as previously described^70^.

Mestrenova version 8.0.1–10878 (Mestrelab Research S.L., Spain) software was used to process and analyze the ^1^H NMR spectra. Equal concentrations of TSP in each sample were used as an internal standard for normalization. The figures show ^1^H NMR data from a minimum of three independent experiments presented as mean ± standard deviation (SD) and statistical significance was calculated with unpaired Student's *t* tests. A *p* value below 0.05 was considered significant.

### Cell viability, apoptosis, and cell cycle assays

Cell viability was assessed as described previously^70^. In brief, the cell number was adjusted to 20,000 cells/ml and triplicates of 100 µl were plated per 96-well. For GLSi treatment, we plated the cells in neurosphere medium containing various drug concentrations (1, 5, 10 µM for C968 and 0.1, 0.5, 1.0 µM for CB839) or vehicle (DMSO). For the rescue experiments cells were treated with 10 µM C968, 1 µM CB839, or equal volumes of DMSO and either 4 mM Glu (Sigma, #G1251–100G) or 4 mM αKG (Sigma, #7589–25G) were added to the different conditions. The viable cell mass was assessed using the CellTiter-Blue® Cell Viability Assay (Promega, #G8081) or Thiazolyl Blue Tetrazolium Bromide (MTT) (Sigma, #2128–1G) according to the manufacturer’s instructions. For CellTiter-Blue® the fluorescence was measured at 560ex/590em and for MTT absorbance it was measured at 570 nm (reference 650 nm) using a Safire 2 multiplate reader (Tecan, Switzerland). Biological replicates analyzed in Fig. 2: *n* = 4 for NSC, JHH520, GBM1, 407 (C968), 268 (C968), and SF188 (C968); *n* = 3 for 233, 407 (CB839), 268 (CB839), and SF188 (CB839).

Apoptosis induction was measured with the “Muse® Annexin V and Dead Cell Assay Kit” (Merck Millipore, USA). Therefore, GSCs were cultured in medium containing 10 µM C968, 1 µM CB839, or vehicle (DMSO) for 48 h, stained for Annexin V and 7-AAD according to the manufacturer’s instructions, and flow cytometry measurements were performed on a Muse® cell analyzer (Merck Millipore) as described in the manufacturer’s instructions.

The cell cycle was analyzed with the “Muse® Cell Cycle Assay Kit” (Merck Millipore). Cells were treated with 10 µM C968, 1 µM CB839, or vehicle (DMSO) for 48 h, fixed with ice cold 70% ethanol at −20 °C for at least three hours and the DNA content was stained according to the manufacturers’ instructions. Flow cytometry measurements were performed on a Muse® cell analyzer (Merck Millipore).

### Clonogenicity assays

The clonogenicity of GSCs was assessed with colony forming assays in semi-solid agarose medium as described previously^70^. In brief, six-well plates were coated with 1.5 ml of 1% agarose (Gibco, #18300012) in pre-warmed neurosphere medium. After 1 h incubation at RT, 2 ml of a single-cell suspension (3000 cells/well) in 0.6% agarose in neurosphere medium was added. After 1 h incubation at room temperature, 2 ml neurosphere medium was added as a top layer. To test the effect of C968 and CB839 on GSC clonogenicity, we either added drugs (10 µM C968 or 1 µM CB839) or equal volumes of vehicle (DMSO) to the upper medium layer. Twice a week the top layer was removed and 2 ml fresh medium (with drug or vehicle) was added. After 3 weeks the top layer was removed, replaced by 1 ml of 1 mg/ml 4-Nitro blue tetrazolium chloride (NBT) (Sigma, #11383213001) in PBS and incubated overnight at 37 °C. The stained colonies were counted using the Clono Counter software^74^. Figure 3 includes the analysis of three biological replicates (*n* = 3) for all cell lines but NSCs. Due to the observed increased degree of biological variations between the replicates, we performed the assay with this cell line more often (*n* = 4 for C968, *n* = 6 for CB839).

### DCFDA ROS assay and GSH/GSSG ratio detection

Accumulation of ROS was measured using 2′,7′-Dichlorofluorescin diacetate (DCFDA) (Sigma, #D6883). Briefly, JHH520, GBM1, 407, and 233 cells were washed with PBS once and resuspended in PBS containing 50 µM DCFDA. After incubation of 30 min at 37 °C, cells were again washed with PBS, resuspended in neurosphere medium, and 3 × 10^5^ cells per condition were transferred into flasks containing either drugs (10 µM C968 or 10 µM CB839) or equal volumes of vehicle (DMSO). The cells were incubated for 24 or 48 h at 37 °C under standard cell culture conditions. As a negative control, cells were incubated with 3 mM of the ROS inhibitor NAC for 24 h, as a positive control, cells were incubated with 500 µM H_2_O_2_ for 30 min. After 24 or 48 h the cells were washed once with PBS and resuspended in 700 µl PBS. For every condition, 200 µl triplicates of each condition were transferred into 96-well microplates. Fluorescence was measured at 493em/515ex on a Safire 2 multiplate reader (Tecan).

For the quantification of the total reduced GSH and GSSG content we used the “GSH/GSSG Ratio Detection Assay Kit” (abcam, #ab138881) according to the manufacturer’s instructions. In brief, GSC cultures were treated with either 10 µM C968 or 1 µM CB839 for 48 h. Subsequently, the total protein content was measured using the “DC™ Protein Assay Kit II” (BioRad, #5000112) according to the manufacturer’s instructions. The cells were lysed in 0.5% NP40 in PBS, centrifuged for 15 min at 13,000 rpm and 4 °C, and the supernatant was used in a deproteinization reaction. Therefore, proteins were precipitated with 4 M phosphochloric acid diluted to a final concentration of 1 M within the cell lysate. After 2 min of centrifugation at 13,000 rpm at 4 °C, the cleared supernatant was then neutralized to pH 4–6 by addition of 2 M KOH. This deproteinized lysate was then analyzed with the above mentioned GSH/GSSG Ratio Detection Assay Kit. The detected reduced and oxidized GSH (nmol) was then normalized to the total protein content (mg) of the respective samples.

### RNA sequencing data from IVY Glioblastoma Project

As described previously^70^ RNA sequencing data were generated from anatomic structures isolated by laser microdissection. Five tumor structures (leading edge *n* = 19, infiltrating tumor *n* = 25, cellular tumor *n* = 112, perinecrotic zone *n* = 27, and pseudopalisading cells around necrosis *n* = 41) were identified by H&E staining and compared with hyperplastic blood vessels (*n* = 23) and the microvascular proliferative region (*n* = 29). A total of 122 RNA samples were generated from 10 tumors and used for sequencing. The data were retrieved in March 2018. Website: ©2015 Allen Institute for Brain Science. Ivy Glioblastoma Atlas Project [Internet]. Available from: glioblastoma.alleninstitute.org.

### Statistical analyses

All statistics were performed with GraphPad Prism Software Version 8.0.2 (GraphPad Software Inc., USA). All results are presented as mean ± SD from a minimum of at least three independent biological replicates. To calculate statistical significance in an experiment with two conditions (treated vs. untreated) we performed two-tailed *t*-tests. When more than two conditions were compared with each other (mRNA expression data) we performed one-way ANOVA analyses and Bonferroni’s tests for multiple comparisons. If applicable, normal distribution was confirmed with the Shapiro–Wilk method. For all experiments, significance was defined as a *p* value below 0.05.

## Supplementary information

Supplementary Figure 1
