# Supplementary figures and images for: A comparative pharmaco-metabolomic study of glutaminase inhibitors in glioma stem-like cells confirms biological effectiveness but reveals differences in target-specificity
Source: Cell Death Discov. 2020 Apr 16;6:20. doi: 10.1038/s41420-020-0258-3 (PMC7162917; doi:10.1038/s41420-020-0258-3)

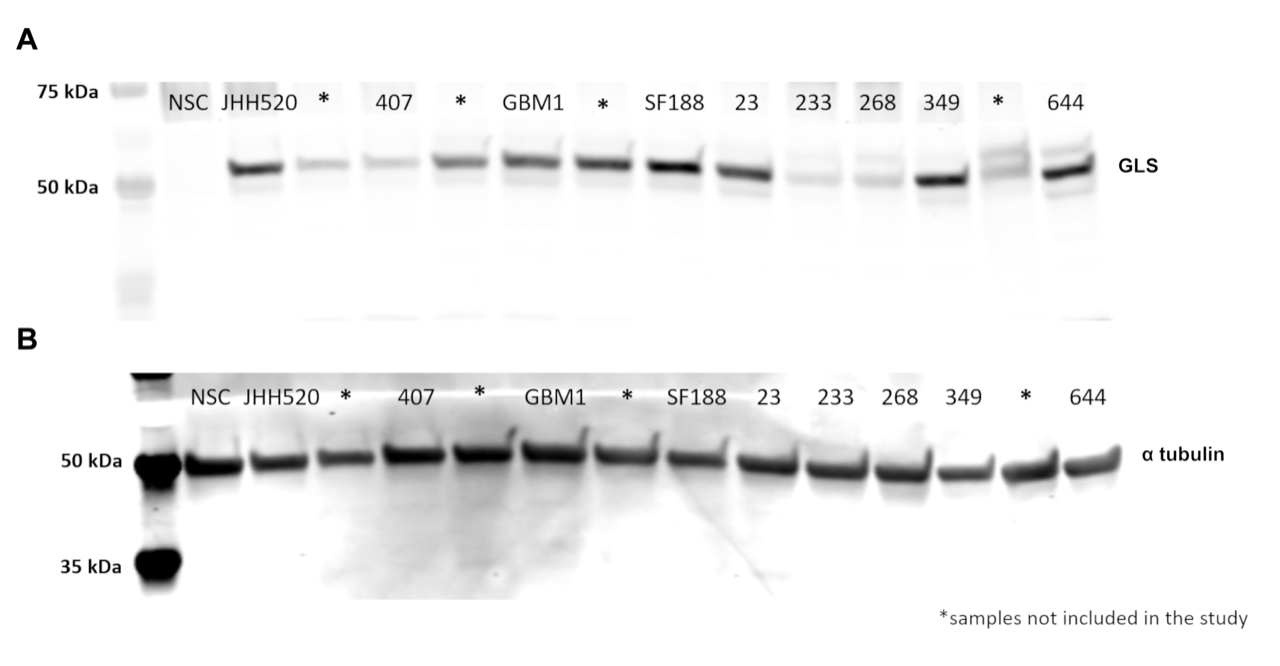

Supplement: Supplementary file 1 — Supplementary Figure 1 [file 41420_2020_258_MOESM1_ESM.tif]
